# Macronutrient Intake and Food Categories’ Contribution to Daily Energy Intake According to BMI in Primary School Children in Croatia

**DOI:** 10.3390/nu16244400

**Published:** 2024-12-21

**Authors:** Lidija Šoher, Daniela Čačić Kenjerić, Martina Pavlić, Ivana Rumbak, Nataša Šarlija, Ana Ilić, Darja Sokolić

**Affiliations:** 1 Department of Food and Nutrition Research, Faculty of Food Technology Osijek, Josip Juraj Strossmayer University of Osijek, Franje Kuhača 18, 31000 Osijek, Croatia; lidija.soher@ptfos.hr; 2 Centre for Food Safety, Croatian Agency for Agriculture and Food, Ivana Gundulića 36b, 31000 Osijek, Croatia; martina.pavlic@hapih.hr (M.P.); darja.sokolic@hapih.hr (D.S.); 3 Laboratory for Nutrition Science, Department of Food Quality Control, Faculty of Food Technology and Biotechnology, University of Zagreb, Pierottijeva 6, 10000 Zagreb, Croatia; ivana.rumbak@pbf.unizg.hr (I.R.); ana.ilic@pbf.unizg.hr (A.I.); 4 Faculty of Economics and Business, Josip Juraj Strossmayer University of Osijek, Trg Ljudevita Gaja 7, 31000 Osijek, Croatia; natasa.sarlija@efos.hr

**Keywords:** school-age children, weight status, dietary patterns, energy intake, food categories

## Abstract

Background: Nutritional status in childhood is associated with a number of short- and long-term health effects. The rising prevalence of childhood obesity highlights the necessity of understanding dietary patterns in children. The study provides an assessment of energy and macronutrient intake and food categories’ contribution to energy intake in Croatian primary school children, according to BMI status. Methods: To assess dietary habits, results of the National Food Consumption Survey on Infants and Children based on EU Menu methodology (OC/EFSA/DATA/2016/02 CT3) were used. The sample included 476 children, aged 6 to under 10 years. Results: Results indicated that one in four children was overweight or obese (27.7%). In total, the mean energy intake was 1598.4 ± 380.3 kcal/day, with 30.7% of the children above the recommended energy intake. Cereals, cereal products, and potato food category were the primary sources of energy, which is in line with the recommendations, with protein and fat intakes exceeding recommended levels. Substantial contribution of sweets and low contribution of fruits and vegetables were observed across all BMI categories, with the difference in energy contribution of fruits (*p* = 0.041) and vegetables (*p* = 0.033). The meat, poultry, fish, and eggs category were the contributors to energy intake from protein and fat, in the majority of BMI groups. Conclusions: In the obese group, higher energy intake from certain food subgroups was recorded, stressing the need for a more detailed dietary assessment. The study’s cross-sectional design limits causal inferences, indicating a need for future longitudinal research to better understand the dynamics of dietary patterns and BMI status in primary school children.

## 1. Introduction

Healthy and adequate nutrition is important in children’s physical growth, cognitive development, and overall health. Every stage of life, from conception to adolescence, has specific nutritional needs [1]. Adequate energy and nutrient intake are crucial during periods of rapid growth [2,3]. Over- and undernutrition in childhood may lead to both short- and long-term consequences. Short-term effects may include academic underperformance and dental problems, while long-term effects can include obesity and other non-communicable diseases [2,4,5,6,7]. Childhood obesity has become one of the biggest public health challenges. In 2007, the WHO Regional Office for Europe established the European Childhood Obesity Surveillance Initiative (COSI), emphasizing the need for standardized surveillance on the prevalence of overweight and obesity among school-aged children. Data from the 2022 COSI report showed that 29% of children in Europe were living with overweight or obesity [8]. The results presented in 2024 showed a somewhat higher trend in Croatia, with 36.1% of eight-year-old children being overweight or obese [9]. Overweight and obese children have a higher risk of being overweight as an adult. Over 60% of children who are overweight before puberty will be overweight in early adulthood [10]. Many developed countries are experiencing an increase in the incidence of overweight, obesity, and chronic diseases across all age groups, largely due to changes in lifestyle patterns, including dietary and physical activity habits [4,5,11]. Overweight and obesity among children are the result of multiple biological, behavioral, and environmental factors that affect long-term energy balance [12]. Throughout childhood, several environmental factors influence the development of dietary habits and food choices. Sociodemographic factors, such as education, parental nutrition knowledge, and family food dynamics have been associated with children’s dietary habits [13,14,15]. The transition from daycare to a school environment brings additional challenges, as children start making more food choices on their own, which makes it a crucial period for establishing healthy eating habits [1,16]. Many school-age children are not meeting their nutritional needs. They often have a low intake of fruits and vegetables and consume unhealthy snacks high in sugar, saturated fat, sodium, and salt [1,13]. Energy intake may play a critical role in childhood obesity. Consistently high-energy intake, often from calorie-dense, nutrient-poor foods, can lead to excessive weight gain and obesity. Therefore, adopting proper dietary habits at an earlier age could play an important role in preventing obesity development later in life [1,4,7,8,17,18]. This further emphasizes the need for consistent dietary habits assessment, especially in vulnerable groups such as school-age children. It provides insights into their nutritional intake, identifying any deficient or excessive intake that may affect growth, development, and long-term health outcomes [7]. 

To implement strategies designed for specific groups and their dietary habits, countries periodically conduct food consumption surveys to gain insight into dietary habits at the population level [5]. The National Food Consumption Survey on Infants and Children in Croatia, conducted according to the EU Menu methodology [19], provides the first harmonized dataset for this population in Croatia. Several studies on this population use small, non-representative samples or focus on specific subgroups. Therefore, this study is significant as it provides the first comprehensive insight into Croatian primary school children’s energy and macronutrient intake, as well as the contribution of different food categories to overall energy intake according to the Body Mass Index (BMI). By evaluating dietary intake and its relationship to BMI, the study fills a gap in existing research and provides a foundation for further research, nutritional interventions, and health policy development.

## 2. Materials and Methods

To assess dietary habits in children from 6 to under 10 years of age, data obtained within the National Food Consumption Survey on Infants and Children based on EU Menu methodology (OC/EFSA/DATA/2016/02 CT3) were used [19]. The survey and methodology of the study were approved by the National Ethical Board of Institute for Medical Research and Occupational Health (RegNo: 100-21/21/18-9) and the Croatian Personal Data Protection Agency (Class: 004-02/17-01/900; RegNo: 567-02/12-18-05). The methodology used in this study has been described in detail elsewhere [20].

### 2.1. Study Population and Data Collection

The National Food Consumption Survey on Infants and Children (OC/EFSA/DATA/2016/02 CT3) was carried out from January 2017 to July 2021. The survey encompassed 1820 children from 6 Croatian regions, of which 322 were infants (from 3 months old to 12 months old), 535 toddlers (from 1 year to 3 years old), and 963 were older children (from 3 years to 9 years old). Based on a database of the Ministry of Internal Affairs, the study population was stratified by region, sex, and age, taking into account equal distribution through four seasons. Potential participants were selected randomly based on set criteria, forming a representative sample. Hospitalized participants, those living in different institutionalized homes, or those living abroad were excluded from the survey. Before the study began, parents or guardians were informed in detail about the course and purpose of the study, after which consent forms were signed.

For the present study, data from 476 children aged 6 to under 10 years were used. Data were collected through one face-to-face in-home interview and two computer-assisted telephone interviews (CATI). An in-home face-to-face interview encompassed gathering anthropometric information, completing a general questionnaire and Food Propensity Questionnaire (FPQ) with information on dietary supplement intake, and instructions on how to complete a food diary. Two CATI interviews served as a control check on the entered dietary consumption data into the software by parents. NutriCro^®^ 2.0 software, which contains all participants’ information, general questionnaires, and food records, was developed especially for this survey and presents a novelty in the food diary application. Apart from general data, the questionnaire collected information on parents’ education, and sociodemographic characteristics, and included a Food Propensity Questionnaire [21] on food and food supplements. Parents’ education level was categorized according to the International Standard Classification of Education (ISCED) as low (early childhood education, primary education, and lower secondary education), medium (upper secondary education and post-secondary non-tertiary education), and high level of education (short-cycle tertiary education, bachelor or equivalent level, master or equivalent level, doctoral or equivalent level). Physical activity level was assessed for children. Parents self-assessed children’s physical activity levels as low, medium, and high.

### 2.2. Anthropometric Measurement and Dietary Intake Assessment

Participants’ weight (kg) and height (cm) were measured on the first face-to-face interview using a digital scale (Seca 877^®^, Hamburg, Germany) and stadiometer (Seca 217^®^, Hamburg, Germany), respectively. Otherwise, anthropometric measurements were recorded by parents/guardians during pediatrician appointments (±two weeks from the interview).

BMI was calculated as weight in kilograms divided by height in squared meters. To determine the nutritional status of children, AntropoPlus [22] and cut-offs for BMI-for-age defined by WHO [23] were used. BMI was categorized as one of the following five categories: severely underweight (<−3 SD), underweight (>−3 SD to ≤−2 SD), normal weight (>−2 SD to ≤1 SD), overweight (>1 SD to ≤2 SD), and obesity (>2 SD).

Dietary consumption was recorded through two non-consecutive food diaries, evenly distributed over days of the week and all four seasons (one year). Keeping food diaries was fully computer-assisted and data were entered directly into NutriCro^®^ 2.0 software. The software used in this study was developed specifically for this study and was further updated after a pilot study to better suit the users, both interviewers and parents/guardians. Each parent/guardian was given detailed instructions on how to collect and enter a food diary. They were instructed to record all foods, drinks, and supplements consumed by the child in quantities in which it was consumed and not to change their usual dietary habits. Apart from quantities, foods were described in detail, including preparation methods, if applicable, and manufacturers. NutriCro^®^ software contains a food consumption database consisting of a national food composition database [24], simple and composite dishes, as well as food available on the market. Determination of portion sizes was also computer-assisted, and a validated picture book [25], several picture sets from the PANCAKE study [26], standard portion sizes, and household measures were incorporated into the software. In the event that none of the above-mentioned options were sufficient, parents/guardians were left with the option of entering free text. Trained interviewers with backgrounds in human nutrition, dietetics, or food science did all data collecting and administering the interviews.

Daily intake of macronutrients, energy, and food categories was calculated on an individual level and presented as an average intake for the group. Average energy and macronutrient intake were compared to the Dietary Reference Values for the EU (DRVs) proposed by the European Food Safety Authority (EFSA) [27]. Energy intake requirements were calculated on an individual level according to age, sex, and physical activity level. According to age, sex, and body mass for protein requirements, population reference intake (PRI) was used. Reference intake range (RI) was also used for assessing the adequacy of fat and carbohydrate intake.

For the purpose of this study, food consumption data were categorized into 10 main food categories and additional subcategories (Table S1). The main food categories were as follows: Grains, grain products, and potatoes; fruit; vegetables; legumes, nuts, and seeds; meat, poultry, fish, and eggs; milk and dairy products; fats and oils; salty snacks; sweets and beverages.

### 2.3. Data Analysis

The sociodemographic and anthropometric characteristics of participants were presented as frequency and percentage or mean and standard deviation (SD) for the categorical and continuous variables, respectively. Dietary intake was presented as mean and SD, followed by the percentage of children above the recommended intake, whereas the contribution of food categories was presented as median and 25th–75th centile.

Normality was tested using the Shapiro–Wilk W test. For comparison between more than two groups, the Kruskal–Wallis ANOVA test was used. As a post hoc test, multiple comparisons of mean ranks for all groups (*p*-values; two-sided significance levels with a Bonferroni adjustment) were used. The level of statistical significance was established at the level of *p* < 0.05. Data processing was performed using the statistical software package Statistica version 14.0.1.25 (1984–2020 TIBCO Software Inc., Hamburg, Germany) and Microsoft Excel 2016 (version 16.0.5413.1000, 2016 Microsoft Corporation, Redmont, WA, USA).

## 3. Results

### 3.1. Sociodemographic and Anthropometric Characteristics

Participants’ sociodemographic and anthropometric characteristics according to their BMI status are presented in Table 1. A total of 476 children, 54.6% boys and 45.5% girls, with a mean age of 7.7 ± 1.1, were included in the present study. According to the BMI, 27.7% of children were overweight or obese with an average z-score of 1.5 ± 0.3 and 2.9 ± 0.8, respectively. Severely underweight were 1.5% and underweight 2.5% of the children included in the study. The Zagreb (28.4%) and Dalmatia regions (28.8%) were the most represented regions in the sample. In the group of overweight children, 35.7% of them came from the Zagreb region, whereas in the obese group, 22.8% came from the Lika and Banovina regions. In total, 52.9%, 44.5%, and 2.5% of the children had medium, high, and low physical activity levels, respectively. In the overweight and obese group, for 2.4% and 3.6% of the children, low physical activity levels were reported. A total of 72.1% of the parents were highly educated and 55% of the households’ monthly incomes were above 1514 €.

### 3.2. Energy and Macronutrient Intake and Adherence to DRVs

According to the BMI status, mean energy and macronutrient intake with the percentage (%) of children above the recommended intake are presented in Table 2. For the mean energy and macronutrient intakes, no significant difference was found among BMI groups. In total, the mean energy intake was 1598.4 ± 380.3 kcal/day, with 30.7% of the children above the recommended energy intake. Overall, mean fat contribution to energy intake is at the upper end of the recommendations, with 52.1% of primary school children above the recommendations. Although not statistically significant, the highest percentage of children (58.3%) above the recommended fat intake (20–35%E) were recorded in the obese group, with a mean fat intake of 65.0 ± 19.2 g/day. Mean carbohydrate intake was 194.2 ± 48.9 g/day, with intakes for the majority of children in the recommended range (45–60%E). In total, 3.8% of the children had carbohydrate intakes above recommendations. Unlike carbohydrate intake, protein intake was above recommendations in 98.1% of the children.

### 3.3. Main Food Categories’ Contribution to Energy Intake and Energy from Macronutrients

**Figure 1:**
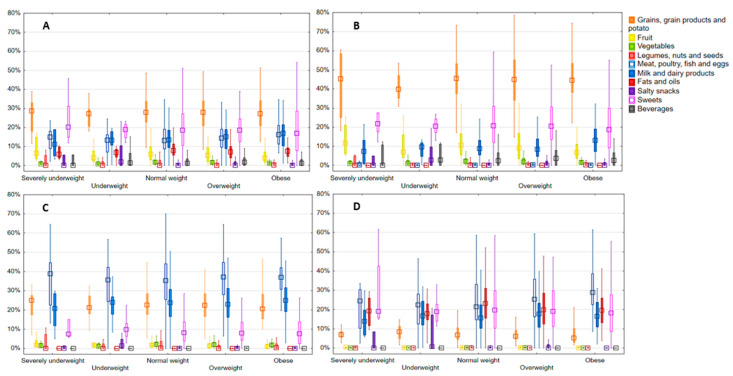
Contribution of main food categories (%) to the total energy intake (**A**), to the energy intake from carbohydrates (**B**), protein (**C**), and fat (**D**) according to BMI status (median; 25th–75th centile).

The main food categories’ contribution to energy intake and energy from macronutrients according to BMI status is illustrated in Figure 1. The contribution is presented as the median and interquartile range (Table S2). Results indicated that the cereals, cereal products, and potato category contributed the most to energy intake, followed by sweets. The third food category in energy contribution, depending on BMI group, was dairy and dairy products, or meat, poultry, fish, and eggs. No significant difference was found according to BMI status in before mentioned categories. Among the main food categories, difference was found in the energy contribution of fruits (*p* = 0.041) and vegetables (*p* = 0.033). The highest contribution of fruit was recorded in the severely underweight (6.35%; 3.48–14.87%), and lowest in the obese group (3.82%; 1.77–7.17%), whereas for vegetables, the highest contribution to energy intake was recorded in the normal weight group (1.66%; 0,89–2.81%), and the lowest in severely underweight (0.77%; 0.55–2.00%). In beverages, no significant difference was found according to BMI status.

Among the main food categories, cereal, cereal products, and potato contributed the most to energy intake from carbohydrates. Statistically significant differences according to BMI status were observed for fruit (*p* = 0.038) and milk and dairy products (*p* = 0.044) for contribution to energy intake from carbohydrates. Milk and dairy products had the highest contribution in the obese group (13.13%; 7.28–19.13%). In contribution to energy intake from protein, no significant differences were found among BMI groups. In all observed BMI groups, meat, poultry, fish, and eggs contributed the most to energy intake from protein. The highest median contribution of the before-mentioned food category was observed in the severely underweight group (38.79%; 22.50–44.57%). In terms of energy intake from fat, a significant difference was found in the meat, poultry, fish, and eggs food category (*p* = 0.026). It contributed the most in the obese (28.80%; 22.49–38.61%), overweight (25.24%; 16.35–35.71%), underweight (22.38%; 12.58–28.01%), and severely underweight (24.40%; 10.34–30.33%) groups, while in the normal weight group (23.03%; 15.63–30.95%), fats and oils contributed the most.

## 4. Discussion

Childhood and adolescent obesity are becoming an increasingly significant health issue [4,5,6,7,8,11]. Despite limiting factors in nutritional assessment, BMI as an anthropometric indicator of nutritional status remains a useful, cost-effective, and simple tool for assessing nutritional status [4]. Using BMI-for-age to determine children’s nutritional status, the results of the presented study showed that 68.3% of children had normal weight and 27.7% were overweight or obese. In comparison, results obtained from three COSI surveys showed a higher prevalence of overweight and obese children in Croatia. The COSI survey results for 2015/2016, 2018/2019, and 2021/2022 showed that 34.9%, 35.0%, and 36.1% of eight-year-olds were overweight or obese, respectively [9]. When it comes to underweight children, similar results were found. The smaller number of participants included in the study, participants’ age, and methodology may account for the observed differences in overweight children. Overall, every fourth child from six to under 10 years of age in Croatia is overweight or obese. Neighboring countries recorded similar results when it comes to children’s BMI status [6,11,28]. In Albania, a study conducted on the population of primary school children found that among first-grade children, 71.2% had normal body weight, while 26.8% had excess body weight, with 14.8% overweight and 12.0% obese [11].

A mean energy intake of 1598.4 ± 380.3 kcal/day is similar to those of 7-year-old Italian children [6], but lower than the results presented by Zeković et al. [28] in the child population (1730.4 ± 470.2 kcal/day). The presented results did not show a statistically significant difference in mean daily energy intake among BMI groups. Morales-Suárez-Varela et al. [4] recorded a significant difference in BMI groups in school-age boys but not in girls. Mean intake in girls with obesity was similar to the underweight one. The absence of the presumed higher energy intake among obese children may also result from parents’ awareness of their children’s food intake and habits, leading to a conscious or unconscious underreporting [29]. However, the fact that 30.7% of children in this study exceeded the recommended energy intake highlights the importance of monitoring and identifying parts of the children’s diet that can be modified. In the present study, when compared with DRVs, the distribution of macronutrient intakes as a percentage of energy showed an imbalance, favoring protein and fats. Similar results regarding an unbalance in the overall diet towards total fats and protein were presented in a few other European studies [4,6,30,31,32]. Out of all the school children in the study of Morales-Suárez-Varela et al. [4], 84.5% presented a lipids intake over the DRVs, whereas our results showed a lower percentage of primary school children with a fat intake over the DRV (52.1%). Although the difference in average fat intake is not significant, it was observed that as BMI increases, so does the percentage of children whose fat intake exceeds the recommended levels. Despite concerns about fat intake contributing to childhood obesity, evidence is mixed. Some studies suggest that higher fat intake is linked to increased body fatness, while others show no such association [32].

With a median contribution to energy intake of 27.9% and 18.4%, the grains, grain products, and potato and the sweets categories, respectively, are food categories that contribute the most to energy intake in Croatian primary school children. In Serbian children, grain and grain products accounted for more than one-third of the total energy intake, whereas sugar and sugar products accounted for 5.4% [28]. Differences in results may come from different ages and food categorization. Research in school-age children in Lebanon showed the same two food categories as main energy contributors, with the sweets, sweetened beverages, and desserts category being the main one, with 21.9% of energy intake [33]. Excessive consumption of energy-dense foods, such as sweetened beverages, may be associated with overweight in children [1,17]. The ToyBox Study that included preschoolers from five European countries showed limited associations between health-related behaviors and BMI, but did find a significant association between higher BMI and higher soft drink consumption in boys [17]. Our findings did not show the difference in contribution to energy intake among BMI groups when it comes to beverages. Although the differences are not statistically significant, there were noticeable differences in contributions to energy intake when it comes to a child’s BMI status. Amongst BMI groups, the highest contribution of sweets was recorded in the severely underweight group. After detailed analysis, the same was noticed for potato-backed snacks in the salty snack category. Although there were few participants in this group, the result might be due to replacing more nutritious food with sweets and snacks as a result of picky eating [34].

A higher intake of snacks and sweets goes hand in hand with a lower intake of fruits and vegetables in the children population [1]. The combined contribution of fruits and vegetables to energy intake (7.04%) is similar to the available results from other European countries. Fruits and vegetables contributed 6% to 9% of energy intake in countries included in the review by McCarthy et al. [35], which may be explained by low energy density and low consumption of this food category. Notably lower contributions to energy intake were recorded compared to the results in Lebanon children aged 9 to 13 years, whose fruit and vegetable contributions were 3.72% and 9.61%, respectively [33]. In the present study, a significant difference in fruit and vegetable contribution to energy intake was observed according to the children’s BMI. The highest contribution of fruit and the lowest contribution of vegetables were recorded in severely underweight children, whereas the lowest contribution of fruit was observed in the group of obese children and the highest vegetable contribution in normal-weight children. A dietary pattern rich in fruits and vegetables is linked to a reduced risk of overweight and obesity in children and adolescents [36]. The results highlight the need to systematically promote the intake of fruits and vegetables in primary school children and indicate that all groups, regardless of BMI status, require attention.

Meat, poultry, fish, and eggs category, and milk and dairy products, occupy the third and fourth places in terms of their contribution to energy intake, depending on BMI status. In the total sample, the reported energy contribution from the food groups mentioned above is higher than that presented by Nasreddine et al. [33] for both food categories. When compared to results in Serbian children, similar results were recorded for the meat category but lower for the milk and dairy [28]. A detailed analysis revealed significant differences in contribution to energy intake in certain food subcategories according to BMI status; processed meats (*p* = 0.004) and flavored milk (*p* = 0.000). The highest contribution for both subcategories was recorded in the obese group. Although milk and dairy products are not associated with obesity in children, flavored milk can be an additional source of added sugar in their diet, contributing to the intake of excess calories [12]. Supporting this, the results demonstrated that according to BMI status when it comes to milk and dairy products the highest contribution to energy intake from carbohydrates was recorded in the obese group. Further analysis showed a significant difference in the subcategory of flavored milk. Overall, when it comes to energy intake from carbohydrates cereal products and potatoes contribute the most to energy intake and intake from carbohydrates, which is in line with the recommendations [37].

A recent meta-analysis showed that higher protein intake, with emphasis on those of animal origin, in children was linked to higher BMI [38]. In addition, a positive association between moderate and high consumption of processed meats and higher BMI was shown in the study by Pozza Santos et al. [29]. The meat, poultry, fish, and eggs category contributed the most to energy intake from protein in all BMI categories, and from fat in almost all BMI groups, suggesting a tendency to consume meat and meat products with higher fat content. The exception is normal-weight children, where the fats and oils category contributed the most to energy intake from fats. Among BMI groups, results showed that children with obesity had the highest energy intake from fat from the meat, poultry, fish, and eggs category. Apart from the significant difference recorded in the main category, the differences were recorded in the subcategories of processed meats (*p* = 0.004) and flavored milk (*p* = 0.000). Processed meats contributed the most in the obese group to energy intake from protein and fat. Meat products such as kulen, sausages, bacon, and other smoked meats have an important part in dietary habits in Croatia due to the long tradition of producing cured meats in households and family farms, which may account for higher consumption of processed meats [39].

It could be hypothesized that childhood obesity is linked to high energy intake and poor diet quality, but several previous studies revealed that there was no significant difference in diet quality between children classified as overweight/obese and their peers with normal weight [17,40,41]. Effects of diet, physical activity levels, and sedentary behavior might not be immediately apparent, especially in younger individuals, but they can have significant impacts on health later in life [1,17,18].

These are among the first results on dietary intake in primary school children in Croatia, according to BMI status, and should be considered in the context of its strengths and limitations. The study followed a standardized methodology [19] and included a representative, stratified sample, which is the main strength. The methodology ensures consistency and reliability of the results, and sample stratification ensures that all subgroups, according to age, sex, or region, are represented. The primary limitation is the cross-sectional nature of the study design. Cross-sectional studies provide an overview of dietary intake at a single point in time, which limits the ability to make causal relationships among variables. Self-reported dietary data, while comprehensive, are at risk of recall bias and underreporting, particularly of socially undesirable foods. The study provides valuable baseline data on dietary intake in Croatia using a robust and standardized methodology. However, the cross-sectional design limits causal conclusions, and future longitudinal studies are needed to monitor the changes in dietary patterns and their connection with BMI status over time. Assessment of the primary contributors to energy intake and energy intake from macronutrients can be used to enhance the effectiveness of obesity prevention initiatives in primary school children.

## 5. Conclusions

This study revealed that one out of four children in primary school in Croatia is overweight or obese. Most of the children had an adequate energy intake, with the grains, grain products, and potato category being the main contributor to energy intake, which aligns with dietary guidelines. Protein and fat intakes exceed the recommendations. Intake of sweets and low consumption of fruits and vegetables were recorded across all BMI categories. Meat, poultry, fish, and eggs contributed significantly to protein and fat intake in most BMI groups. Additionally, children with obesity showed higher energy intake from specific food subgroups, which needs further research.

Results provide valuable insight into primary school-age children’s food consumption and could be used to model initiatives aimed at promoting healthy dietary habits in primary school children in Croatia. A detailed analysis of food categories and specific regions could provide additional insights into which dietary aspects should be targeted in different regions of Croatia regarding children’s BMI status.

## Figures and Tables

**Table 1:** Participants’ sociodemographic and anthropometric characteristics according to BMI-for-age status.

	Total (*N* = 476)	Severely Underweight (*n* = 7; 1.5%)	Underweight (*n* = 12; 2.5%)	Normal Weight (*n* = 325; 68.3%)	Overweight (*n* = 84; 17.6%)	Obese (*n* = 48; 10.1%)
Sex, *n* (%)						
Boys	260 (54.6)	4 (57.1)	8 (66.7)	165 (50.8)	53 (63.1)	30 (62.5)
Girls	216 (45.4)	3 (42.9)	4 (33.3)	160 (49.2)	31 (36.9)	18 (37.5)
Age, mean (sd)	7.7 (1.1)	7.6 (0.7)	7.2 (1.1)	7.7 (1.1)	7.8 (1.1)	7.9 (1.0)
*n* (%)						
6 y	146 (30.7)	1 (14.2)	6 (50.0)	103 (31.7)	24 (28.6)	12 (25.0)
7 y	131 (27.5)	3 (42.9)	3 (25.0)	81 (24.9)	27 (32.1)	17 (35.3)
8 y	130 (27.3)	3 (42.9)	2 (16.7)	93 (28.6)	19 (22.6)	13 (27.1)
9 y	69 (14.5)	0 (0.0)	1 (8.3)	48 (14.8)	14 (16.7)	6 (12.5)
Antropometric characteristics, mean (sd)						
Height (cm)	131.6 (8.8)	137.6 (9.1)	125.8 (5.9)	130.7 (8.4)	133.5 (8.8)	135.0 (10.6)
Weight (kg)	29.2 (7.4)	21.9 (2.7)	20.0 (2.0)	26.7 (4.5)	33.6 (5.9)	41.8 (8.9)
BMI z-score	0.32 (1.4)	−3.6 (0.7)	−2.5 (0.3)	−0.17 (0.8)	1.5 (0.3)	2.9 (0.8)
Season, *n* (%)						
Spring	109 (22.9)	3 (42.8)	3 (25.0)	74 (22.8)	16 (19.0)	13 (27.1)
Summer	110 (23.1)	1 (14.3)	3 (25.0)	81 (24.9)	17 (20.2)	8 (16.7)
Fall	149 (31.3)	2 (28.6)	2 (16.7)	94 (28.9)	35 (41.8)	16 (33.3)
Winter	108 (22.7)	1 (14.3)	4 (33.3)	76 (23.4)	16 (19.0)	11 (22.9)
Region, *n* (%)						
Dalmatia region	88 (18.4)	1 (14.3)	3 (25.0)	59 (18.2)	19 (22.6)	6 (12.5)
Istria, Primorje and Gorski kotar regions	42 (8.8)	0 (0.0)	0 (0.0)	35 (10.8)	4 (4.8)	3 (6.3)
Lika and Banovina regions	40 (8.4)	1 (14.3)	1 (8.3)	21 (6.4)	6 (7.2)	11 (22.8)
Northern Croatia region	75 (15.8)	0 (0.0)	1 (8.3)	55 (16.9)	9 (10.7)	10 (20.8)
Slavonia region	96 (20.2)	0 (0.0)	3 (25.0)	68 (20.9)	16 (19.0)	9 (18.8)
Zagreb region	135 (28.4)	5 (71.4)	4 (33.4)	87 (26.8)	30 (35.7)	9 (18.8)
Physical activity level, *n* (%)						
High	212 (44.5)	1 (14.3)	3 (25.0)	152 (46.8)	40 (47.6)	16 (33.3)
Medium	252 (52.9)	6 (85.7)	8 (66.7)	167 (51.4)	42 (50.0)	29 (60.4)
Low	12 (2.5)	0 (0.0)	1 (8.3)	6 (1.8)	2 (2.4)	3 (6.3)
Parents education level *, *n* (%)						
High	343 (72.1)	6 (85.7)	11 (91.7)	238 (73.2)	62 (73.8)	26 (54.2)
Medium	122 (25.6)	1 (14.3)	1 (8.3)	80 (24.6)	21 (25.0)	19 (39.5)
Low	11 (2.3)	0 (0.0)	0 (0.0)	7 (2.2)	1 (1.2)	3 (6.3)
Household income/month, *n* (%)						
Not reported	46 (9.7)	0 (0.0)	1 (8.3)	34 (10.5)	7 (8.3)	4 (8.3)
<378 €	1 (0.2)	0 (0.0)	0 (0.0)	0 (0.0)	1 (1.2)	0 (0.0)
378–757 €	18 (3.8)	0 (0.0)	0 (0.0)	9 (2.8)	4 (4.8)	5 (10.4)
757–1141 €	51 (10.7)	1 (14.3)	3 (25.0)	31 (9.5)	8 (9.5)	8 (16.7)
1141–1513 €	98 (20.6)	1 (14.3)	2 (16.7)	66 (20.3)	14 (16.7)	15 (31.3)
>1513 €	262 (55.0)	5 (71.4)	6 (50.0)	185 (56.9)	50 (59.5)	16 (33.3)

* International Standard Classification of Education (ISCED): Low education–ISCED levels 0–2; medium education—ISCED levels 3–4; high education—ISCED levels 5–8.

**Table 2:** Mean energy and macronutrient intake with the percentage of children above the recommended intake.

	DRV Value	Total (*N* = 476)	Severely Underweight (*n* = 7; 1.5%)	Underweight (*n* = 12; 2.5%)	Normal Weight (*n* = 325; 68.3%)	Overweight (*n* = 84; 17.6%)	Obese (*n* = 48; 10.1%)	*p* Value **
**Energy** * (kcal/day)	1312–2165	1598.4 ± 380.3	1565.8 ± 269.8	1606.6 ± 399.7	1607.8 ± 386.7	1564.4 ± 389.5	1595.9 ± 336.8	0.858
*% above DRV*		*30.7*	*28.6*	*33.3*	*32.0*	*22.0*	*29.2*	
**Total fat** (g/day)		63.7 ± 21.5	59.9 ± 18.3	63.9 ± 21.1	63.9 ± 21.5	62.4 ± 23.4	65.0 ± 19.2	0.870
%E	20–35%E	35.4 ± 6.2	33.9 ± 6.7	35.5 ± 6.1	35.3 ± 6.2	35.5 ± 6.8	36.3 ± 5.4	
*% above DRV*		*52.1*	*42.9*	*41.7*	*51.4*	*53.6*	*58.3*	
**Carbohydrates** (g/day)		194.2 ± 48.9	192.8 ± 35.3	199.1 ± 55.3	195.9 ± 49.6	189.3 ± 50.1	190.7 ± 42.3	0.893
%E	45–60%E	48.9 ± 6.5	49.6 ± 6.9	49.8 ± 7.0	49.0 ± 6.5	48.6 ± 7.3	48.0 ± 5.4	
*% above DRV*		*3.8*	*0.0*	*0.0*	*4.3*	*4.8*	*0.0*	
**Proteins** * (g/day)	0.89–0.92 g/kg bw per day	55.8 ± 14.1	56.7 ± 11.9	52.6 ± 14.5	56.1 ± 14.6	55.2 ± 13.8	55.6 ± 11.3	0.957
*% above DRV*		*98.1*	*100*	*100*	*99.4*	*98.8*	*91.7*	

* Adherence to recommendations was assessed at the individual level, according to age, sex, and physical activity for energy, and body mass. ** Kruskal–Wallis ANOVA test; level of statistical significance at *p* < 0.05. Percentage (%) of participants above the DRV Value in italic.

## Data Availability

The data are available from the Croatian Agency for Agriculture and Food, but restrictions apply to the availability of these data. Data are available upon request and with permission of the Croatian Agency for Agriculture and Food.

## Supplementary Materials

The following supporting information can be downloaded at: https://www.mdpi.com/article/10.3390/nu16244400/s1, Table S1: Food categories and subcategories, Table S2: Contribution of food categories (%) to the total energy intake and to energy intake from macronutrients according to BMI.
