# Supplementary material for: Macronutrient Intake and Food Categories’ Contribution to Daily Energy Intake According to BMI in Primary School Children in Croatia
Source: Nutrients. 2024 Dec 21;16(24):4400. doi: 10.3390/nu16244400 (PMC11679920; doi:10.3390/nu16244400)
Supplement: Supplementary file 1 [file nutrients-16-04400-s001.zip › nutrients-3361461-supplementary.pdf]

Table S1 Food categories and subcategories

Food category	Food subcategory
Grains, grain products and potato	*Bread, rolls and tortillas*
	Grains, grits and flour
	Pasta
	Breakfast cereals
	Fresh and frozen potatoes and tubers
Fruit	*Fresh and frozen fruits*
	100% fruit juices
	Canned fruits
	Dried fruits and fruit bars (100% fruits)
Vegetables	*Fresh and frozen vegetables*
	Canned and dried vegetables
Legumes, nuts and seeds	*Fresh, dried, canned and frozen legumes*
	Nuts without added salt and sugar
	Seeds and seed products
	Milk and meat alternatives
	Fermented milk alternatives
Meat, poultry, fish and eggs	*Eggs*
	Fresh and frozen red meat
	Fresh and frozen poultry
	Fresh and frozen fish, seafood and products
	Processed meats (red meat and poultry)
	Frozen breaded meat and fish products
Milk and dairy products	*Milk*
	Fermented dairy products
	Cheese and cheese spreads
	Flavored milk
	Flavored fermented dairy products
	Milk desserts
	Infant formulas
Fats and oils	*Plant oils*
	Animal fats
Salty snacks	*Grain based salty snacks*
	Potato based salty snacks
Sweets	*Chocolate and chocolate spreads with additives*
	Cocoa powder
	Biscuits and dry cakes
	Cakes and tarts
	Marmalades, jams and jellies
	Honey and sweeteners
	Ice creams based on water and/or milk
	Sugar
	Confectionery and chewing gum
	Other
Beverages	*Water*
	Tea
	Fruit/vegetable juices
	Other non-alcoholic beverages

Table S2 Contribution of food categories (%) to the total energy intake and to energy intake from macronutrients according to BMI status

	**Total (N=476)**	**Severely underweight (n=7; 1.5%)**	**Underweight (n=12; 2.5%)**	**Normal weight (n=325; 68.3%)**	**Overweight (n=84; 17.6%)**	**Obese (n=48; 10.1%)**	**p value***
A Contribution of food categories (%) to the total energy intake *Median (25th-75th centile)*							
Grains, grain products and potato	27.90 (22.13 - 33.66)	28.61 (17.92 - 33.01)	27.26 (20.60 - 29.56)	27.97 (22.89 - 33.67)	27.87 (20.88 - 33.93)	27.27 (20.93 - 34.18)	0.894
Fruit	5.55 (2.66 - 9.41)[ab]	6.35 (3.48 - 14.87)[ab]	4.15 (2.33 - 7.73)[ab]	5.81 (3.02 - 9.79)[a]	5.28 (2.10 - 9.51)[ab]	3.82 (1.77 - 7.17)[b]	0.041
Vegetables	1.49 (0.79 - 2.65)	0.77 (0.55 - 2.00)	0.79 (0.49 - 1.94)	1.66 (0,89 - 2.81)	1.47 (0.80 - 2.72)	1.17 (0.69 - 1.77)	0.033
Legumes, nuts and seeds	0.12 (0.00 - 2.22)	0.00 (0.00 - 5.38)	0.03 (0.00 - 2.11)	0.16 (0.00 - 2.84)	0.00 (0.00 -1.24)	0.27 (0.00 - 1.44)	0.212
Meat, poultry, fish and eggs	13.36 (9.04 - 19.40)	14.83 (9.03 - 18.07)	13.33 (7.06 - 16.25)	13.03 (8.71 - 19.15)	14.29 (9.61 - 19.08)	16.26 (11.24 - 21.01)	0.070
Milk and dairy products	13.98 (9.41 - 18.86)	11.17 (5.08 - 18.82)	13.35 (10.78 - 17.80)	13.53 (9.51 - 18.50)	15.03 (8.90 - 18.97)	16.99 (10.36 - 21.49)	0.244
Fats and oils	7.56 (4.86 - 10.45)	5.83 (3.83 - 9.80)	6.81 (4.37 - 8.57)	7.95 (5.43 - 11.22)	7.13 (4.01 - 10.12)	7.49 (4.70 - 9.39)	0.211
Salty snacks	0.00 (0.00 - 2.09)	0.00 (0.00 - 5.45)	1.90 (0.00 - 10.31)	0.00 (0.00 - 1.25)	0.00 (0.00 - 1.99)	0.00 (0.00 - 1.18)	0.075
Sweets	18.40 (10.74 - 27.10)	20.24 (14.94 - 31.11)	18.95 (14.74 - 21.68)	18.45 (10.66 - 27.20)	18.49 (12.49 - 24.54)	16.86 (7.84 - 28.64)	0.815
Beverages	1.26 (0.00 - 3.49)	0.00 (0.00 - 5.45)	1.35 (0.00 - 6.36)	1.18 (0.00 - 3.44)	1.77 (0.00 - 4.13)	1.25 (0.00 - 3.16)	0.692
B Contribution of food categories (%) to the energy intake from carbohydrates *Median (25th-75th centile)*							
Grains, grain products and potato	44.99 (36.64 - 53.47)	45.36 (24.95 - 58.48)	39.84 (35.19 - 47.11)	45.51 (37.43 - 53.16)	44.86 (34.10 - 55.29)	44.63 (38.30 - 53.52)	0.777
Fruit	9.82 (4.78 - 16.33)[ab]	11.73 (6.11 - 20.98)[ab]	6.89 (4.09 - 15.98)[ab]	10.65 (5.54 - 16.60)[a]	9.18 (3.59 - 16.71)[ab]	6.69 (3.72 - 11.20)[b]	0.038
Vegetables	1.93 (0.99 - 3.41)	0.92 (0.80 - 2.37)	1.02 (0.62 - 2.66)	2.12 (1.07 - 3.61)	1.70 (0.97 - 3.68)	1.58 (0.81 - 2.36)	0.053
Legumes, nuts and seeds	0.04 (0.00 - 1.49)	0.00 (0.00 - 4.88)	0.01 (0.00 - 0.49)	0.07 (0.00 - 1.63)	0.00 (0.00 - 0.81)	0.18 (0.00 - 1.42)	0.397
Meat, poultry, fish and eggs	0.17 (0.00 - 0.47)	0.19 (0.06 - 1.08)	0.05 (0.00 - 0.25)	0.16 (0.00 - 0.44)	0.24 (0.00 - 0.58)	0.16 (0.00 - 0.48)	0.398
Milk and dairy products	8.99 (5.51 - 13.80)[ab]	7.37 (0.94 - 13.16)[ab]	9.70 (4.72 - 11.81)[ab]	8.73 (5.49 - 13.12)[a]	8.40 (4.85 - 13.82)[ab]	13.13 (7.28 - 19.13)[b]	0.044
Fats and oils	0.00 (0.00 - 0.00)	0.00 (0.00 - 0.00)	0.00 (0.00 - 0.00)	0.00 (0.00 - 0.00)	0.00 (0.00 - 0.00)	0.00 (0.00 - 0.00)	0.612
Salty snacks	0.00 (0.00 - 1.99)	0.00 (0.00 - 4.93)	2.74 (0.00 - 9.75)	0.00 (0.00 - 1.21)	0.00 (0.00 - 2.17)	0.00 (0.00 - 1.47)	0.068
Sweets	20.67 (12.30 - 30.65)	21.86 (17.64 - 27.58)	20.50 (17.33 - 24.35)	20.79 (12.02 - 31.05)	20.48 (13.57 - 30.69)	18.72 (9.26 - 30.00)	0.827
Beverages	2.61 (0.00 - 6.79)	0.00 (0.00 - 10.84)	2.86 (0.00 - 11.05)	2.48 (0.00 - 6.54)	3.69 (0.00 - 7.80)	2.67 (0.00 - 6.61)	0.734
C Contribution of food categories (%) to the energy intake from protein *Median (25th-75th centile)*							
Grains, grain products and potato	22.17 (17.22 - 28.26)	24.82 (17.40 - 27.51)	21.16 (17.99 - 27.34)	22.56 (17.60 - 28.46)	22.38 (16.63 - 28.44)	20.52 (15.50 - 28.06)	0.680
Fruit	1.56 (0.44 - 2.86)	1.78 (1.01 - 4.33)	1.73 (0.94 - 2.45)	1.69 (0.66 - 2.96)	1.48 (0.07 - 2.60)	0.96 (0.00 - 2.27)	0.094
Vegetables	2.00 (1.01 - 3.37)	1.44 (0.69 - 2.82)	1.13 (0.53 - 2.65)	2.08 (1.08 - 3.48)	1.95 (1.01 - 3.34)	1.82 (0,65 - 2.65)	0.288
Legumes, nuts and seeds	0.18 (0.00 - 3.15)	0.00 (0.00 - 7.28)	0.07 (0.00 - 2.47)	0.30 (0.00 - 3.82)	0.00 (0.00 - 1.78)	0.44 (0.00 - 2.46)	0.229
Meat, poultry, fish and eggs	35.51 (26.54 - 44.20)	38.79 (22.50 - 44.57)	35.57 (24.46 - 42.14)	35.16 (25.41 - 44.07)	37.12 (27.87 - 44.72)	36.90 (30.63 - 41.91)	0.467
Milk and dairy products	23.66 (16.81 - 30.60)	20.65 (11.85 - 28.43)	23.90 (17.85 - 26.84)	23.65 (16.65 - 30.60)	22.90 (16.27 - 31.08)	24.94 (19.11 - 31.45)	0.691
Fats and oils	0.00 (0.00 - 0.08)	0.00 (0.00 - 0.03)	0.00 (0.00 - 0.06)	0.00 (0.00 - 0.10)	0.00 (0.00 - 0.07)	0.00 (0.00 - 0.04)	0.383
Salty snacks	0.00 (0.00 - 0.86)	0.00 (0.00 - 1.43)	1.35 (0.00 – 4.83)	0.00 (0.00 - 0.59)	0.00 (0.00 - 0.89)	0.00 (0.00 - 0.56)	0.117

Sweets	8.08 (4.06 - 13.85)	7.51 (6.26 - 15.02)	9.88 (6.29 - 13.14)	8.23 (3.86 - 13.96)	7.99 (4.13 - 13.69)	7.60 (2.54 - 13.87)	0.816
Beverages	0.00 (0.00 - 0.00)	0.00 (0.00 - 0.00)	0.00 (0.00 - 0.00)	0.00 (0.00 - 0.00)	0.00 (0.00 - 0.16)	0.00 (0.00 - 0.01)	0.370
D Contribution of food categories (%) to the energy intake from fat<br>*Median (25th-75th centile)*							
Grains, grain products and potato	6.66 (4.31 - 10.29)	7.04 (5.54 - 8.62)	8.46 (5.00 - 11.30)	6.79 (4.46 - 10.48)	6.06 (4.57 – 9.32)	5.25 (2.87 - 10.12)	0.554
Fruit	0.20 (0.00 - 0.49)	0.49 (0.18 - 0.64)	0.15 (0.00 - 0.48)	0.20 (0.00 - 0.49)	0.23 (0.00 - 0.49)	0.00 (0.00 - 0.38)	0.254
Vegetables	0.17 (0.05 - 0.48)	0.13 (0.02 - 0.33)	0.09 (0.02 - 0.26)	0.20 (0.06 - 0.52)	0.16 (0.06 - 0.57)	0.13 (0.04 - 0.27)	0.215
Legumes, nuts and seeds	0.02 (0.00 - 0.43)	0.00 (0.00 - 0.70)	0.00 (0.00 - 0.30)	0.04 (0.00 - 0.62)	0.00 (0.00 - 0.26)	0.06 (0.00 - 0.26)	0.218
Meat, poultry, fish and eggs	23.05 (15.20 - 33.72)^{ab}	24.40 (10.34 - 30.33)^{ab}	22.38 (12.58 - 28.01)^{ab}	21.30 (14.77 - 32.98)^{a}	25.24 (16.35 - 35.71)^{ab}	28.80 (22.49 - 38.61)^{b}	0.026
Milk and dairy products	15.88 (10.63 - 22.70)	13.95 (6.86 - 19.65)	16.64 (12.10 - 24.22)	15.66 (10.37 - 22.40)	17.66 (10.91 - 23.15)	16.36 (11.10 - 23.66)	0.882
Fats and oils	21.30 (14.42 - 29.85)	19.18 (11.64 - 25.96)	17.82 (14.81 - 22.86)	23.03 (15.63 - 30.95)	19.78 (12.49 - 28.39)	19.56 (13.26 - 25.89)	0.077
Salty snacks	0.00 (0.00 - 1.35)	0.00 (0.00 - 8.55)	0.72 (0.00 - 17.10)	0.00 (0.00 - 0.56)	0.00 (0.00 - 1.95)	0.00 (0.00 - 0.20)	0.083
Sweets	19.06 (10.68 - 28.74)	18.91 (15.70 - 42.45)	18.74 (14.07 - 22.79)	19.61 (10.37 - 29.73)	18.92 (11.22 - 29.88)	18.19 (8.61 - 27.56)	0.708
Beverages	0.00 (0.00 - 0.00)	0.00 (0.00 - 0.00)	0.00 (0.00 - 0.00)	0.00 (0.00 - 0.00)	0.00 (0.00 - 0.08)	0.00 (0.00 - 0.00)	0.272

**Kruskal-Wallis ANOVA test; $^{a, b}$ Multiple comparisons of mean ranks for all groups (p-values; two-sided significance levels with a Bonferroni adjustment) post hoc test; level of statistical significance at p<0.05*
